# Serum Vitamin D as a Biomarker in Autoimmune, Psychiatric and Neurodegenerative Diseases

**DOI:** 10.3390/diagnostics12010130

**Published:** 2022-01-06

**Authors:** Giulia Bivona, Caterina Maria Gambino, Bruna Lo Sasso, Concetta Scazzone, Rosaria Vincenza Giglio, Luisa Agnello, Marcello Ciaccio

**Affiliations:** 1 Department of Biomedicine, Neurosciences and Advanced Diagnostics, Institute of Clinical Biochemistry, Clinical Molecular Medicine and Clinical Laboratory Medicine, University of Palermo, 90127 Palermo, Italy; giulia.bivona@unipa.it (G.B.); cmgambino@libero.it (C.M.G.); bruna.losasso@unipa.it (B.L.S.); concetta.scazzone@unipa.it (C.S.); giglio.rosaria.vincenza@gmail.com (R.V.G.); luisa.agnello@unipa.it (L.A.); 2 Department of Laboratory Medicine, University Hospital “P. Giaccone”, 90127 Palermo, Italy

**Keywords:** vitamin D, biomarker, autoimmune diseases, neurodegenerative diseases, psychiatric diseases, Alzheimer’s disease, multiple sclerosis, standardization, 25(OH)D

## Abstract

Vitamin D is a steroid hormone regulating calcium-phosphorus homeostasis, immune response and brain function. In the past thirty years, an increasing number of cohort studies, meta-analyses and randomized controlled trials (RTCs) evaluated the serum levels of 25-hydroxyvitamin D [25(OH)D], which is considered the Vitamin D status biomarker, in patients affected by neurological, psychiatric and autoimmune diseases. Although an association between low 25(OH)D serum levels and the prevalence of these diseases has been found, it is still unclear whether the serum 25(OH)D measurement can be clinically useful as a biomarker for diagnosis, prognosis and predicting treatment response in neurodegeneration, mental illness and immune-mediated disorders. The lack of standardized data, as well as discrepancies among the studies (in the analytical methods, cut-offs, endpoints and study sets), weakened the findings achieved, hindered pooling data, and, consequently, hampered drawing conclusions. This narrative review summarizes the main findings from the studies performed on serum 25(OH)D in neurological, psychiatric and autoimmune diseases, and clarifies whether or not serum 25(OH)D can be used as a reliable biomarker in these diseases.

## 1. Introduction

Vitamin D is an endogenous compound belonging to the steroid hormones class.

Humans synthesize active Vitamin D starting from a precursor on the skin, although a small amount can also be taken by diet.

Vitamin D became known in the early 1900s, and its metabolism was broadly revised when it was found in a wide range of organs, tissues and cytotypes producing the hormone and receiving its signal. It was born in the era of the so-called Vitamin D “non-skeletal activities”, with an increasing number of studies uncovering some roles of the hormone, counting the regulation of brain function and immune response [1]. In addition, the autocrine and paracrine fashion in which Vitamin D behaves in these organs and tissues became apparent. Since Vitamin D has been regarded as a neurosteroid and immunomodulator, the 25(OH)D circulating levels in patients affected by neurological, psychiatric and autoimmune diseases have been evaluated [2,3,4,5,6]. The rationale to search for a role of Vitamin D in these diseases was that the hormone can influence and modify various cerebral processes, including the effectiveness of connectivity in some neural circuits involved in cognition, memories and emotional behavior. Additionally, it can modulate many immunological events of both innate and adaptive arms of immunity, ranging from neutrophils and macrophages’ antimicrobial activity to T helper lymphocytes cytokine production. Consistently, Vitamin D circulating levels have been extensively measured in patients affected by autoimmune diseases, revealing, for instance, a large spectrum of *Vitamin D receptor* (VDR) polymorphisms in the frequency and severity of some pathologies, and a high prevalence of low Vitamin D concentrations among these patients. Additionally, a broad number of authors have focused their research on the impact of low Vitamin D levels on some features of neurodegenerative diseases, specifically Alzheimer’s disease (AD) and Parkinson’s disease (PD), uncovering a possible role of the hormone as a biomarker for predicting disease severity in these patients.

Collectively, in the past three decades, a wide amount of studies have been per-formed on the 25-hydroxyvitamin D [25(OH)D] levels in patients having neurological, psychiatric and autoimmune diseases, but these studies were quite different in terms of patients, design, methods, assay technologies, and displaying diverse strengths and flaws. On the one hand, this could explain the failure to reach univocal conclusions on a possible role of serum 25(OH)D as a biomarker for the hormone in such diseases, and, on the other hand, this represents a rationale to review the literature on this topic, in order to synthesize and critically analyze the main findings and conclusions from the studies.

This narrative review summarizes the main findings from the studies performed on Vitamin D serum levels in patients affected by the autoimmune, neurological and psychiatric diseases, and clarifies whether 25(OH)D measurement in these patients impacts their clinical management, influencing diagnosis, prognosis and treatment of these patients.

## 2. Vitamin D Metabolism

**Figure 1:**
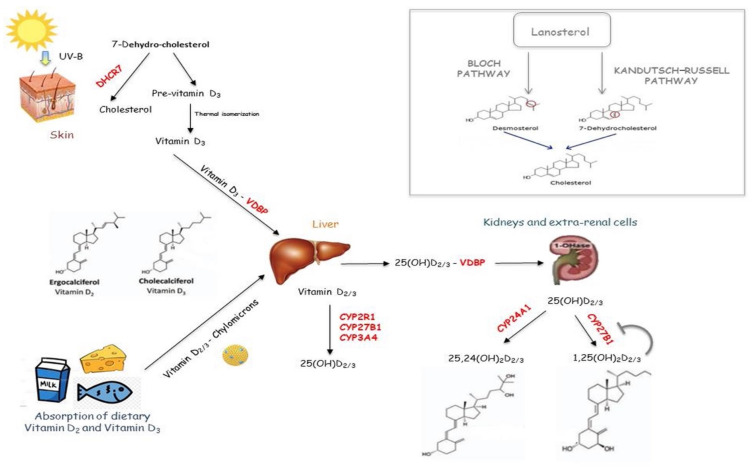
Vitamin D metabolism pathway. Vitamin D active form is produced in a multi-step process, including the ultraviolet B (UVB) rays’ irradiation of a cutaneous precursor [7-dehydro-cholesterol (7-DHC)], and two hydroxylation steps. In addition to the synthesis of Vitamin D, 7-DHC can be oriented toward the production of cholesterol by Kandutsch–Russell biochemical pathway.

The Vitamin D active form is produced in a multi-step process, including the ultraviolet B (UVB) rays’ irradiation of a cutaneous precursor [7-dehydro-cholesterol (7-DHC)], and two hydroxylation steps. In addition to the synthesis of Vitamin D, 7-DHC can be oriented toward the production of cholesterol by the 7-DHC reductase (7-DHCR) enzyme, within the Kandutsch–Russell biochemical pathway, that occurs in the skin, brain, muscle and heart (Figure 1) [1]. Since 7-DHCR regulates the amount of DHC available for Vitamin D conversion, this enzyme represents the first limiting step for Vitamin D synthesis.

UVB irradiation of 7-DHC forms the cholecalciferol, which undergoes first hydroxylation in the liver by a 25 hydroxylase (CYP2R1, CYP3A4 and CYP27A1), generating 25(OH)D, and the second in the kidney by renal 1,25 hydroxylase (CYP27B1), producing 1,25, dihydroxyvitamin D [1,25(OH)2D]. The latter is released in blood to reach the bowel, where it regulates calcium absorption. However, 1,25 hydroxylase is present within various organs and cells, including the lung, brain, prostate, placenta and immune system cells, where the active Vitamin D can be synthesized to regulate some cellular processes, including cell differentiation and proliferation [7]. Renal CYP27B1 is up-regulated by the parathyroid hormone (PTH) and down-regulated by the fibroblast growth factor (FGF23) and 1,25(OH)2D, whereas extra-renal CYP27B1 is regulated by interferon γ (IFN-γ) and tumor necrosis factor (TNF) [7,8].

Vitamin D binding protein (VDBP) transports both 25(OH)D and 1,25(OH)2D from the liver and kidney to other tissues, where they are taken up throughout the plasma membrane by HSP70 [1,9]. VDBP is a high-polymorphic gene, and, as a consequence, there is wide variability in the protein function. As a result, the individual’s response to Vitamin D supplementation varies according to the VDBP variants, influencing the amount of circulating exogenous protein-bound Vitamin D (see Section 5) [9]. Within the megalin cubilin complex- non-expressing cells, 25(OH)D reaches mitochondria by HSP70, where it is converted in 1,25(OH)2D. Then, it can be transported to the nucleus, where it binds the Vitamin D receptor (VDR) [10], leading to the genomic and non-genomic actions (for more details on Vitamin D genomic and non-genomic actions see refs [11,12]). Cell-specific factors, including transcription factors, can influence how a citotype responds to the VDR/ligand binding. Thus, different tissues display distinct feedbacks to the hormone since the CYP27B1 promoter region responds to different stimuli based on the cell types [1,13]. For instance, 1,25(OH)2D inhibits CYP27B1 in the renal cells, but not in macrophages.

Vitamin D catabolism is carried out by a 24 hydroxylase enzyme (CYP24A1).

**Figure 2:**
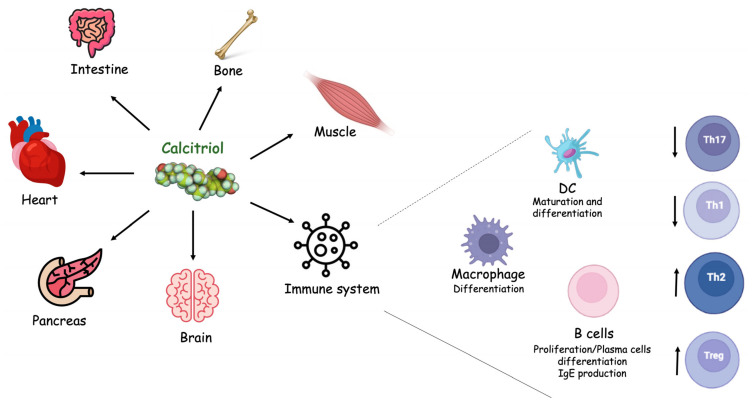
Vitamin D biological functions. Vitamin D’s active form, also known as calcitriol, exerts pleiotropic functions on different cells and organs.

Vitamin D exerts pleiotropic effects. The most known function of Vitamin D is the regulation of calcium homeostasis. However, over the course of the last decades, it has become increasingly clear that the effects of Vitamin D are not limited to the maintenance of calcium homeostasis. Indeed, it regulates multiple cellular processes, including cell growth and differentiation, and influences the functions of cells of the immune, nervous and cardiovascular systems as well as muscles and the pancreas (Figure 2). The role of Vitamin D in inflammation has gained attention. First of all, immune cells produce 1,25(OH)2D and express the VDR and the enzymes for metabolizing Vitamin D3. The Vitamin D3 synthesized within CNS exerts both autocrine and paracrine immunomodulating effects on innate and adaptive immune responses. Specifically, it promotes the anti-inflammatory phenotype by regulating T cells, B cells and antigen-presenting cells (dendritic cells and macrophages) (Figure 2).

## 3. Vitamin D Status Assessment and 25(OH)D Measurement Standardization

Vitamin D status is typically evaluated by measuring serum 25(OH)D [14], but it should be highlighted that serum 25(OH)D measurement for many decades has been affected by a lack of standardization.

Standardization means aligning laboratories and assays with the “true” concentration, based on internationally recognized reference procedures and materials, regardless of the place, time and assay systems [15]. To address the problem of 25(OH)D measuring standardization, the Vitamin D Standardization Program (VDSP) has been created, leading researchers working worldwide to collaborate with well-established health institutions (including the International Federation of Clinical Chemistry and Laboratory Medicine-IFCC, the Ghent University, the Vitamin D External Quality Assessment Scheme-DEQAS, and the National Institute for Standard and Technology-NIST). The specific purpose of VDSP is to encourage laboratories and manufacturers to reduce the analytical variability of the 25(OH)D measurement with national health and nutrition surveys [16]. The VDSP protocols for standardization of serum 25(OH)D have been applied to national surveys in Europe, Canada and the USA [17,18,19].

Within the VDSP, the DEQAS aims specifically to assess the accuracy of the results produced by participants. The NIST has developed a liquid chromatography-tandem mass spectrometry (LC/MS-MS) assay, which has been accepted by the Joint Committee for Traceability in Laboratory Medicine (JCTLM) as the reference measurement procedure (RMP) for 25(OH)D [20]. The accuracy of the results is assessed by comparing them to the NIST value, being that the NIST value is recognized as the target value [21,22]. The accuracy of a result is calculated as the percentage of bias of the result from the target (or “true”) value provided by NIST [22].

VDSP collaboration is based on the encouragement to use methods and materials traceable to the NIST reference measurement procedures (RMPs) and standard reference materials (SRMs) [16,21,22,23,24].

As for many other molecules, the lack of standardization in the past decades hindered reaching a consensus on which 25(OH)D levels define Vitamin D sufficiency, deficiency and insufficiency [25,26,27]. Optimal Vitamin D status is regarded as the 25(OH)D levels required to maintain skeletal health, whereby 25(OH)D values below 30 nm/L are considered to be associated with an increased risk of rickets/osteomalacia, while values between 30 and 50 nm/L are considered to be sufficient for skeletal health [15].

## 4. Vitamin D as a Disease Marker

Generally, a biomarker is regarded as a molecule that is used to identify, monitor and predict treatment responses to a certain disease or condition. Specifically, a biomarker is measured to diagnose a disease and to predict its severity, having, consequently, a strong impact on clinical management as per the ward to be oriented and therapy to be administered. Probably, the most requested feature of a biomarker is predicting treatment response, with several known examples on this topic, including procalcitonin for guiding therapy in sepsis. It is widely accepted that the construct of the “clinical usefulness” of a biomarker fully depends on the above-mentioned characteristics. However, it should be noted that a correlation between the marker and some disease features, including severity, does not always mean the biomarker is useful in the clinical practice, because many molecules can change among different disease stages independently of the pathophysiological link between biomarker levels variation and disease features. In other words, before any biomarker enters clinical practice, an influence on treatment administration and response, as well as disease course and patient condition should be proven. Regarding the possible role of Vitamin D as a biomarker in neurological, psychiatric and autoimmune diseases, a correlation between the hormone concentrations and some disease characteristics has been proven. However, it is not clear whether measuring 25(OH)D can modify clinical management and disease course in these patients.

### 4.1. Autoimmune Diseases

Among all autoimmune diseases, 25(OH)D serum levels have been mostly investigated in systemic lupus erythematosus (SLE), systemic sclerosis (SS), rheumatoid arthritis (RA), autoimmune thyroid diseases and multiple sclerosis (MS) (Table 1).

Low Vitamin D levels in patients affected by SLE have been found [28,29,30,31,54,55,56,57,58,59,60], although evidence on the correlation between Vitamin D and disease activity is controversial, as well as the association with inflammatory cytokines and SLE serology [32,33]. A recent meta-analysis highlighted some SLE features that have been associated with Vitamin D deficiency, counting organ damage, SLE flares, neurological and renal involvement, thrombocytopenia, leukopenia, proteinuria, use of corticosteroids and hydroxychloroquine. It is important to note that the authors reported strong heterogeneity among the studies reviewed, which compromised the data analysis [34].

Low Vitamin D levels in SS patients have been documented as well [61,62,63,64]. Some authors reported that the disease severity was not associated with the extent of Vitamin D deficiency and suggested that the decrease in Vitamin D blood levels does not accelerate the worsening of the disease course [35]. Other authors confirmed these findings, excluding a correlation between Vitamin D levels and SS clinical and laboratory findings (i.e., disease duration, systemic involvement, autoantibodies) [12,35,36].

Many studies assessed Vitamin D levels in RA patients, but disputed results have been reported [37,38,39,40,41]. A systematic review and meta-analyses on 3489 patients reported an inverse correlation between 25(OH)D levels and disease activity, which was stronger in studies from developing and low-latitude countries [42]. Some studies documented no significant differences between the RA patients and controls in terms of their 25(OH)D levels, and the correlation between 25(OH)D levels and the RA disease activity and joint damage, in these studies, has not been proven. However, it should be noted that these studies had a small sample size [37,38].

Regarding Vitamin D status among autoimmune thyroid disease patients, multiple lines of evidence documented an association between low Vitamin D levels and disease onset [43,45]. However, other studies showed no significant differences in 25(OH)D levels between autoimmune thyroid disease patients and controls [46,47,48]. The relationship between Vitamin D status and the regulation of TSH, thyroid hormones, autoantibodies and immunological markers, including pro-inflammatory interleukins, is still debated. Furthermore, the clinical usefulness of serum 25(OH)D in predicting prognosis in these patients is controversial, and data on Vitamin D as a predictor of treatment response are limited.

In the end, low Vitamin D levels have been reported in patients affected by MS in both neonatal and adult cohorts [49,50,51,52,53,65,66,67,68,69,70,71,72,73,74,75,76,77]. Munger et al. measured serum 25(OH)D in more than 1000 women from the Finnish Maternity Cohort (FMC), finding that low 25(OH)D levels (<30 nmol/L) were associated with a higher MS risk, compared with normal levels (≥50 nmol/L). However, 25(OH)D levels were measured using a chemiluminescence assay [49], which is not an NIST-recommended RMP. Ascherio et al. performed 25(OH)D determinations in patients enrolled in the BENEFIT study, a large multi-center randomized trial. The authors showed that serum Vitamin D biomarker is a strong risk factor for long-term MS activity and progression in the early disease course, and it is able to predict new active lesions and relapse rate [50]. Collectively, it cannot be excluded that low Vitamin D levels could play a role in the early management of MS patients.

### 4.2. Alzheimer’s Disease and Parkinson’s Disease

AD and PD represent the most common neurodegenerative diseases worldwide. Accordingly, the role of Vitamin D has been widely investigated in patients affected by these diseases.

Vitamin D serum levels in AD patients have been largely explored [78,79,80,81,82,83,84,85,86,87,88,89,90,91,92,93,94,95,96,97]. Findings from a prospective study including 1658 individuals revealed that subjects having 25(OH)D insufficiency had a two-fold risk of AD onset, compared to those having Vitamin D sufficiency [98]. It should be noted that the authors carried out 25(OH)D measurements using RMPs and SRMs, which strengthens to the results. Similar findings have also been described by many studies [99,100,101,102,103]. In 2017, Licher et al. found that Vitamin D serum 25(OH)D concentrations below 25 nmol/L were associated with a higher incidence of AD [99]. Although a large sample size reinforced the study results (more than 3800 subjects), a chemi-luminescence assay was used for determination, achieving not standardized data as the results. In a large meta-analysis, Balion et al. reported an association between Vitamin D serum levels and AD development risk, but revealed remarkable discrepancies among the studies reviewed, highlighting that findings from these studies are weakened by differences in the assay methods and cut-offs used [103].

Many studies failed to demonstrate a correlation between Vitamin D and AD as well [104,105,106,107,108,109]. Olsson et al. [105] reported opposite findings in 1182 men, finding no association between 25(OH)D levels and the risk of AD. It is important to note that NIST-certified assay methods were used to determine 25(OH)D serum levels by Olsson.

Karakis et al. reported no association between Vitamin D levels and incidence of AD in the Framingham Heart Study participants [106]. Although the 25(OH)D measurement in this study was performed by a competitive protein-binding immunoassay, the large sample size and long follow-up period conferred significance to the results. In line with the findings of Olsson and Karakis, Duchaine et al. reported no association between 25(OH)D levels and AD in 660 subjects from the Canadian Study of Health and Aging, although chemiluminescence assay methods were used for this study [107]. Surprisingly, the authors found that increased 25(OH)D concentrations were slightly associated with a higher risk of developing AD in women but not in men and suggested that further investigations are needed to look into the relationship with sex. Other authors confirmed that no relation exists between plasma 25(OH)D concentrations and the incidence of all-cause dementia or AD [108]. Another meta-analysis performed by Jayedi et al. on 27,000 individuals showed that Vitamin D insufficiency and deficiency are not correlated with the risk of developing AD [109] and confirmed that 25(OH)D levels >35 ng/mL could increase AD risk.

Collectively, these findings could not be adequately homogeneous and robust to support a role for 25(OH)D as a biomarker in AD. Additionally, well-established biomarkers have been validated for AD diagnosis and prognosis and are successfully used in clinical practice [110,111,112,113].

Some studies have reported low Vitamin D serum levels among PD patients [114,115,116]. One longitudinal, long-term follow-up study was performed on >3100 subjects by Knekt et al. in 2010 [117]. The authors reported the use of SRM certified by NIST, and findings suggested that 25(OH)D could predict the risk of PD development [117]. However, the small number of PD cases in the sample (*n* = 50) and the possible presence of residual confounders limited the strengths of the findings achieved. Other authors documented a significant association between 25(OH)D serum levels and disease severity [118,119], and two recent and extensive meta-analyses confirmed these results [120,121]. It should be noted that strong discrepancies were reported among the studies in the assay methods used, which requires interpreting findings from these meta-analyses with a grain of salt.

### 4.3. Psychiatric Diseases

Many studies documented an inverse association between 25(OH)D serum levels and major depression disorder (MDD) [122,123,124,125,126,127], although opposite findings have been reported as well [84,85,86,87,88].

Data from the European Male Ageing Study (EMAS), involving 3151 men, revealed that serum 25(OH)D levels were significantly lower (*p* < 0.001) in depressed men compared with non-depressed participants [122], and this association remained significant after adjusting for age, alcohol consumption, physical activity, BMI, physical function and comorbidities. The Korean Urban Rural Elderly (KURE) study was performed on 2942 participants and showed that serum 25(OH)D concentrations were inversely associated with the presence of depressive symptoms in men, but not in women [125]. Despite such evidence, several authors failed to prove the association between serum 25(OH)D levels and MDD [126,127,128,129,130,131,132].

Several authors have proved an association between low serum 25(OH)D levels and schizophrenia, and the relationship between disease severity and Vitamin D circulating levels has also been documented [133,134,135]. Nonetheless, opposite findings have been also reported [136]. A recent meta-analysis on 12,528 participants assessed the relationship between schizophrenia and Vitamin D levels, concluding that schizophrenia onset is more likely among patients with Vitamin D deficiency/insufficiency compared to controls [137]. In addition, cross-sectional studies and those involving outpatients showed significant differences of mean Vitamin D serum levels between schizophrenic patients and controls more frequently than case-control studies and those performed on inpatient populations [137]. However, many drawbacks affected the studies reviewed (selection bias, observational study set, presence of confounders, lack of information on patients’ baseline characteristics, differences among the cut-offs used to define Vitamin D insufficiency and deficiency), leading to a need to interpret findings with caution. Table 2 summarizes the main studies performed on psychiatric disorders.

## 5. Studies on Vitamin D Supplementation

A debated matter is whether low Vitamin D levels could be a modifiable risk factor for neurological and autoimmune diseases. Interventional studies are the most suitable to assess whether any molecule or its deficiency can expose a patient to the onset of a certain disease. To establish whether the optimal Vitamin D status could prevent the onset or modify the course of neurological, psychiatric and autoimmune diseases, supplementation studies have been performed.

In autoimmune diseases patients, Vitamin D supplementation has been proven to ameliorate RA symptoms, such as pain, but evidence on a beneficial effect of Vitamin D supplementation in these patients is limited and needs to be further confirmed [138,139,140]. Some authors reported cholecalciferol supplementation to be effective in decreasing disease activity and reducing fatigue in juvenile SLE patients [141,142]. However, other studies documented a modest beneficial effect of supplementation in improving disease activity in adult SLE patients. Regarding the effect of Vitamin D supplementation in MS patients, a recent meta-analysis summarized findings from six RCTs to assess the impact of Vitamin D administration on the severity and progression of MS, as defined according to the Expanded Disability Status Scale (EDSS) [143]. The authors showed that different dosages and formulations of Vitamin D did not affect EDSS, compared to the placebo. Similar findings were achieved by Zheng et al. in an earlier meta-analysis demonstrating that Vitamin D administration had no effect on MS severity and progression according to the EDSS score and the annual relapse rate (ARR) [144]. Recently, Quirant-Sánchez et al. showed that a combined therapy based on the use of Vitamin D3 and tolerogenic dendritic cells (tolDC) plus interferon beta in a preclinical model of MS ameliorated the disease course compared to each monotherapy. Thus, a combined therapy based on antigen-specific VitD3-tolDC and IFN-beta could represent a promising strategy for MS [145].

In neurodegenerative diseases, questionable results have been gained as well. Some authors evaluated the effect of Vitamin D supplementation in preventing the onset of AD, achieving controversial evidence [146,147]. It should be noted that studies proving an impact of Vitamin D supplementation in cognition had limited populations and follow-up duration; therefore, no clear evidence of a significant influence of low Vitamin D levels on the risk of cognitive decline onset has been earned. Further, a recent RCT performed in critically ill adults with Vitamin D deficiency confirmed that supplementation does not improve cognition and executive function [148].

Likewise, data on the impact of Vitamin D supplementation in preventing PD onset are inconclusive and few, with evidence proving that supplementation has no beneficial effect on motor function in PD patients [121,144]. In 2019, Zhou et al. reported findings from a systematic review documenting no beneficial influence in motor function in PD patients after Vitamin D supplementation. However, the study displayed some drawbacks, counting a small number of study participants and short-term follow-up, which may have affected the results [121]. Oppositely, other authors reported that Vitamin D administration had an effect on the strength and postural balance in these patients, and, interestingly, an impact of Vitamin D supplementation has been documented on disease progression in PD patients carrying CT and TT genotypes of *FokI*, a *VDR* gene polymorphism [149,150,151,152].

A critical matter regarding Vitamin D supplementation is that a remarkable difference among individuals exists as per the response to Vitamin D analogues. Such a difference mainly depends on *VDBP* genetic variants, which sharply influence the 25(OH)D circulating levels, with VDBP being the Vitamin D carrier protein. For instance, Al Daghri et al. reported two single nucleotide polymorphisms (SNPs), rs4588 and rs7041, increasing the risk of non-response to Vitamin D supplementation, regardless of the administered doses [153].

## 6. Conclusions

Vitamin D is one of the most studied vitamins worldwide and its biological activity in humans is still a captivating topic. Low Vitamin D levels occur among neurological, psychiatric and autoimmune patients, but the significance of such findings is far from being clear, either because low Vitamin D levels are common among healthy subjects, or because studies display several drawbacks that sharply weaken the significance of the results achieved. The main pitfalls can be summarized in two different categories: (i) analytical items, concerning laboratory choices of the assay methods used for 25(OH)D measurement, and (ii) methodological issues, regarding the conceptualization and design of the study. From a strictly laboratory perspective, the former affects the reliability and reproducibility of the 25(OH)D measurement, and refers to the standardization issue, which has been deeply faced in the past decades without full success. In this regard, it should be taken into account that standardized assay methods are often expensive and not rapid-to-perform, which hampers their use among healthcare systems and research laboratories. Regarding methodological issues, the discrepancies in the cut-offs used made it difficult to pool data, to the extent that most of the meta-analyses reviewed in the current article suggested interpreting findings with caution. In addition, the study set had a major role in weakening the significance of the results in some cases; for instance, short follow-up period longitudinal studies are not suitable for difficult-to-diagnose diseases such as AD.

The current review has some limitations as well, including that it is not a systematic review and it takes into account only a part of the autoimmune, psychiatric and neurodegenerative diseases. Despite such limitations, it points out that Vitamin D’s role as a biomarker in these diseases, based on the studies reviewed, has not been proven. Although some authors reported that serum 25(OH)D is able to facilitate diagnosis and prognosis in AD, PD and some autoimmune pathologies, no evidence supports its use as a candidate marker for entering clinical practice. Additional concerns have been raised considering that autoimmune and neurodegenerative diseases already benefit from established biomarkers.

Advancing knowledge in this field means correcting analytical and methodological issues, providing 25(OH)D standardized data and producing meta-analyses with robust data and significance. The main goal for future investigations in this scenario is to address the following question: can 25(OH)D measurement have an influence on the clinical management of psychiatric patients, or in autoimmune and neurodegenerative ones?

Another big challenge in this area is around the supplementation of Vitamin D. In AD patients, it is not clear yet how and if Vitamin D appropriate status can help prevent disease onset or ameliorate disease features, despite the relatively large amount of studies that have been performed. Generally, interventional studies are arduous to perform compared to the observational ones, due to high costs, ethic concerns, long time span and other elements. In addition, some apparent difficulties affect RCTs on Vitamin D supplementation, including the toxicity of Vitamin D analogues and the variability of the response due to genetic factors. The main goal for future investigations would be a sort of mapping of the factors influencing the effectiveness of supplementation, to identify patients having benefits from Vitamin D analogues’ administration. This could help move the knowledge on Vitamin D toward clinical practice real-world needs.

## Figures and Tables

**Table 1:** Major findings from the observational studies evaluating Vitamin D serum levels in autoimmune diseases.

Disease	Authors	Ref	Publication Time	Sample Size	Cut-Off Value for Vitamin D	Ethnic/Cultural/Geographical Features	Findings
SLE							
	Petri et al.	[28]	2013	1006	40 ng/mL	Caucasian (54%), African—American (37%), other (8%)	25(OH)D levels of 40 ng/mL or above are not associated with improvement in disease activity
	AlSaleem et al.	[29]	2015	28	Normal ≥30–100 ng/mL, Insufficient 21–29 ng/mL, Deficient <20 ng/mL	Saudi	High daily Vitamin D3 supplementation could impact disease activity
	Eloi et al.	[30]	2017	199	Insufficient 10–30 ng/mL, Deficient <10 ng/mL	Brazilian	Disease activity is associated with lower serum concentrations of 25(OH)D
	Salman-Monte et al.	[31]	2016	102	NA	Spanish	Vitamin D insufficiency is highly prevalent among female SLE patients, even in southern regions
	Andreoli et al.	[32]	2012	115	Insufficient 10–30 ng/mL, Deficient <10 ng/mL	Italian	Patients with antiphospholipid syndrome have low 25(OH)D levels
	Aranow et al.	[33]	2015	125	20 ng/mL	NA	Vitamin D3 supplementation does not diminish the expression of IFNα inducible genes
	Dall’Ara et al.	[34]	2018	NA	NA	NA	Vitamin D insufficiency is endemic in SLE patients
SSc							
	Lin et al.	[35]	2017	554	Insufficient 10–30 ng/mL, Deficient <10 ng/mL	NA	SSc patients exhibit low Vitamin D levels. The severity of clinical features is not associated with the extent of Vitamin D deficit
	Gupta et al.	[36]	2018	38	Normal 30–100 ng/mL, Insufficient 10–30 ng/mL, Deficient <10 ng/mL	NA	Geographic origin or clinical presentation of SSc patients does not influence 25(OH)D levels
RA							
	Polasik et al.	[37]	2017	35	20 ng/mL	Polish	25(OH)D levels were similar in RA patients and age- and gender-matched healthy controls and were not associated with joint damage and disease activity in patients
	Wong et al.	[38]	2017	77	20 ng/mL	Malaysian	Mean 25(OH)D levels in RA patients were low, but similar to the age-matched healthy controls
	Kerr et al.	[39]	2011	850	Insufficient <30 ng/mL, Deficient <20 ng/mL	Caucasian males	25-OH-D insufficiency is associated with disease severity, not clinical disease activity
	Pakchotanon et al.	[40]	2016	239	Normal >30 ng/mL, Insufficient 10–30 ng/mL, Deficient <10 ng/mL	Thai	No associations between serum 25(OH)D levels and disease activity or functional status
	Gopal et al.	[41]	2019	100	50 nmol/L	Malaysian	25(OH)D is not associated with disease activity or serum IL-6 levels but it may have a role in functional disability
	Lin et al.	[42]	2016	3489	NA	NA	Serum Vitamin D levels are lower in RA patients and are inversely associated with disease activity
Autoimmune Thyroiditis							
	Wang et al.	[43]	2015	1782	20 ng/mL	NA	Low levels of serum 25(OH)D are related to autoimmune thyroiditis
	Muscogiuri et al.	[44]	2016	50	50 nmol/L	Italian	Low levels of 25(OH)D were significantly associated with autoimmune thyroiditis in women with polycystic ovary syndrome
	Muscogiuri et al.	[45]	2016	168	Normal ≥20 ng/mL, Deficient ≤20 ng/mL	Italian	Vitamin D deficiency was significantly associated with autoimmune thyroiditis in the elderly
	D’Aurizio et al.	[46]	2015	NA	NA	NA	Conflicting results have been obtained about the association between Vitamin D and autoimmune thyroiditis
	Effraimidis et al.	[47]	2012	78	Insufficient <30 ng/mL, Deficient <20 ng/mL	Caucasian	Vitamin D deficiency is not related to early stages of autoimmune thyroiditis
	Botelho et al.	[48]	2018	159	30 ng/dL	NA	Lower levels of Vitamin D have not been associated with autoimmune thyroiditis
MS							
	Munger et al.	[49]	2006	515	25 nmol/L	White American, Black and Hispanic American	High circulating levels of Vitamin D are associated with a lower risk of MS
	Munger et al.	[50]	2017	1092	Normal >50 nmol/L, Insufficient 30–50 nmol/L, Deficient <30 nmol/L	Finnish females	Vitamin D deficiency is a risk factor for MS
	Nielsen et al.	[51]	2017	521	30 nmol/L	Danish	Low concentrations of neonatal Vitamin D are associated with an increased risk of MS
	Ascherio et al.	[52]	2014	468	50 nmol/L	NA	Higher serum 25(OH)D levels robustly predicted a lower degree of MS activity, brain atrophy and clinical progression over the 5 years of follow-up
	Salzer et al.	[53]	2012	192	75 nmol/L	NA	Association between high 25(OH)D levels during the years preceding disease onset and a decreased risk of MS

NA, not applicable.

**Table 2:** Major findings from the observational studies evaluating Vitamin D serum levels in psychiatric disorders.

Disease	Authors	Ref	Publication Time	Sample Size	Cut-Off Value for Vitamin D	Ethnic/Cultural/Geographical Features	Findings
**MDD**							
	Lee et al.	[122]	2011	3369	Sufficient >75 nmol/L Sub-optimum 50–74.9 nmol/L Insufficient 25–49.9 nmol/L Deficient <25 nom/L	Caucasian	An inverse association between 25(OH)D levels and depression, largely independent of several lifestyle and health factors
	Kjaergaard et al.	[123]	2012	357	NA	Norway	Low levels of serum 25(OH)D were associated with depressive symptoms, but no effect was found with Vitamin D supplementation.
	Milaneschi et al.	[124]	2014	2386	Optimal >50 nmol/L Insufficient 25–50 nmol/L Deficient <25 nmol/L	Netherlands	Low levels of 25(OH)D were associated with the presence and severity of depressive disorder
	Song et al.	[125]	2016	2853	NA	Korea	Lower concentrations of Vitamin D are independently associated with depressive symptoms
	Sherchand et al.	[126]	2018	300	Insufficient 20–29 ng/mL Sufficient 30–100 ng/mL Deficient <20 ng/mL	Nepal	Vitamin D deficient people have increased odds of having clinically significant depression
	Vidgren et al. 2018	[127]	2018	1602	NA	Finland	Lower concentration of serum 25(OH)D is associated with a higher prevalence of depression in an elderly general population
	Zhao et al.	[128]	2010	3916	NA	USA	No association between serum concentrations of 25(OH)D and the presence of moderate-to-severe depression, major depression or minor depression
	Kwasky et al.	[129]	2014	77	NA	USA	No association between Vitamin D and depression
	Can et al.	[130]	2017	175	NA	Turkey	No relationship between depression, Vitamin D levels and Fok 1 polymorphism of Vitamin D receptor.
	Bossola et al.	[131]	2010	80	NA	Italy	No association between Vitamin D and symptoms of depression as well as anxiety in chronic hemodialysis patients
	Almeida et al.	[132]	2015	3105	<50 nmol/L	Australia	The results do not support a role for Vitamin D in the causation of depression
**Schizophrenia**							
	Berg et al.	[133]	2010	1179	Insufficient/deficient < 50 nmol/L	Norway and immigrants	25-hydroxyvitamin D deficiency is common in patients with psychotic disorders
	Crews et al.	[134]	2013	69	Insufficient 25–50 nmol/L Deficient <25 nmol/L	England	Vitamin D levels are low at the onset of a first psychotic episode
	Firth et al.	[135]	2018	2612	NA	Spain, Turkey, India, Poland, China, UK, USA, Pakistan, Singapore, Nigeria, Romania, Norway	Deficits in Vitamin D previously observed in long-term schizophrenia appear to exist from illness onset, and are associated with worse symptomology
	Norelli et al.	[136]	2010	60	Deficient <20 ng/mL Insufficient 20–29 ng/mL Optimal >30 ng/mL	USA	High prevalence rates of Vitamin D deficiency in the general population, including patients with schizophrenia-spectrum disorders regardless of acute care or long-stay inpatient status.
	Zhu et al.	[137]	2020	12,528	NA	Africa, America, Caucasian, Japan, Germany, Spain, Turkey, Pakistan	Vitamin D deficiency is associated with schizophrenia

NA, not available.

## Data Availability

Not applicable.
